# Using Machine Learning to Identify Factors Affecting Antibody Production and Adverse Reactions After COVID-19 Vaccination

**DOI:** 10.3390/vaccines14020115

**Published:** 2026-01-26

**Authors:** Nahomi Miyamoto, Tohru Yamaguchi, Yoshinori Tamada, Seiya Yamayoshi, Koichi Murashita, Ken Itoh, Seiya Imoto, Norihiro Saito, Tatsuya Mikami, Shigeyuki Nakaji

**Affiliations:** 1 Research Institute of Health Innovation, Hirosaki University, 5 Zaifu-cho, Hirosaki-shi 036-8562, Aomori, Japan; nmiyamoto3155@hirosaki-u.ac.jp (N.M.); murasita@hirosaki-u.ac.jp (K.M.); 2 Research Center for Health-Medical Data Science, Hirosaki University Graduate School of Medicine, 5 Zaifu-cho, Hirosaki-shi 036-8562, Aomori, Japan; yamaguchi.tohru@hirosaki-u.ac.jp; 3 Division of Virology, Institute of Medical Science, University of Tokyo, 4-6-1 Shirokanedai, Minato-ku, Tokyo 108-0071, Japan; yamayo@ims.u-tokyo.ac.jp; 4 The University of Tokyo Pandemic Preparedness, Infection and Advanced Research Center, 4-6-1 Shirokanedai, Minato-ku, Tokyo 108-0071, Japan; 5 The Research Center for Global Viral Diseases, National Institute of Global Health and Medicine, Japan Institute for Health Security, 1-21-1 Toyama, Shinjuku-ku, Tokyo 162-86551, Japan; 6 Department of Stress Response Science, Biomedical Research Center, Hirosaki University Graduate School of Medicine, 5 Zaifu-cho, Hirosaki-shi 036-8562, Aomori, Japan; itohk@hirosaki-u.ac.jp; 7 Division of Health-Medical Intelligence, Human Genome Center, Institute of Medical Science, University of Tokyo, 4-6-1 Shirokanedai, Minato-ku, Tokyo 108-0071, Japan; imoto@ims.u-tokyo.ac.jp; 8 Department of Clinical Laboratory Medicine, Hirosaki University Graduate School of Medicine, 5 Zaifu-cho, Hirosaki-shi 036-8562, Aomori, Japan; norihiro@hirosaki-u.ac.jp; 9 Department of Preemptive Medicine, Innovation Center for Health Promotion, Hirosaki University Graduate School of Medicine, 5 Zaifu-cho, Hirosaki-shi 036-8562, Aomori, Japan; tmika@hirosaki-u.ac.jp (T.M.); nakaji@hirosaki-u.ac.jp (S.N.)

**Keywords:** COVID-19 vaccine, SARS-CoV-2 virus, carrier molecules, Iwaki Health Promotion Project, machine learning, Bayesian network

## Abstract

**Background:** Coronavirus disease 2019 (COVID-19) vaccines deliver mRNA packaged in lipid nanoparticles via intramuscular injection. This study investigated several factors influencing antibody production patterns and adverse reactions after vaccination with COVID-19 vaccines. **Methods:** Among the participants of the Iwaki Health Promotion Project (IHPP), 211 individuals who consented to this study were surveyed regarding antibody titers and adverse reaction symptoms following vaccination. A machine learning approaches such as ridge regression, elastic-net, light gradient boosting, and neural network were applied to extract the variables, and Bayesian network analysis was applied to explore causal relationships between health data and the multi-omics dataset obtained from the IHPP health checkups. **Results:** Females with lower levels of free testosterone experienced more adverse reactions than males. Moreover, the immune system is more active in younger individuals, causing adverse reactions and higher antibody production. The Spikevax vaccine induced adverse reaction symptoms with higher antibody production in cases of fever. Meanwhile, drinking 2–3 cups of green tea daily seemed to be effective in increasing antibody production. Factors increasing side effect risk include blood natural killer cell count and muscle quality in the vaccinated arm. Plasma metabolome metabolite concentrations, tongue coating bacterial colonization, and folate intake were also identified as factors influencing side effect risk. Furthermore, characteristics of participants at risk for fever symptoms included longer telomere length, higher antibody production patterns, and higher CD4-positive T cell counts. **Conclusions:** Further investigation of these identified influencing factors is expected to clarify the rationale for new vaccine development and identify lifestyle and dietary habits that enhance vaccine efficacy.

## 1. Introduction

Severe acute respiratory syndrome coronavirus 2 (SARS-CoV-2) caused the coronavirus disease 2019 (COVID-19) pandemic, acquiring various amino acid mutations in the spike protein that amplified its global spread [1,2]. Several measures have been implemented to combat the COVID-19 pandemic, and several measures were implemented [3,4]. In particular, vaccines against COVID-19 were widely recommended in numerous countries [5,6,7,8,9]. In Japan, the COVID-19 vaccines Comirnaty from Pfizer Inc. [10], Spikevax from Moderna Inc. [11], and Vaxzevria from AstraZeneca PLC [12] were approved as medical drugs since 2021. Many reports have since elucidated the relationship between vaccination and the antibody production mode [13,14], adverse reaction symptoms after vaccination [15,16,17,18], and the efficacy of boosters [19,20].

Although messenger RNA (mRNA) vaccines against COVID-19 effectively elicit the production of neutralizing antibodies, various adverse reactions have been reported [21]. mRNA vaccines are injected intramuscularly and taken up by dendritic cells, which induce acquired immune responses against the modified spike proteins [5,22]. Most adverse reactions are likely caused by the lipid nanoparticle (LNP) carrier molecules [23].

The mRNA structures in Comirnaty and Spikevax are similar; however, their LNP components are different [5]. Therefore, the occurrence of adverse reactions varies because of the number of vaccinations or combinations of vaccine types administered. In addition, adverse reactions may depend on the genomic background and lifestyle-related factors of the host, such as dietary habits, which could modulate antibody production. However, few studies have investigated the relationship between antibody production or adverse reactions and the combination of vaccine types or lifestyle-related factors.

Since 2005, the Iwaki Health Promotion Project (IHPP) has been continuously implemented to promote the health of residents of the Iwaki District of Hirosaki City, Aomori Prefecture. Residents of the Aomori Prefecture, located in northern Japan, are known to have a shorter life expectancy and a higher incidence of cancer and lifestyle-related diseases than those in other regions of Japan [24]. Health checkups are an activity of the IHPP for community-dwelling residents. A distinction between the IHPP health checkups is that more than 4000 health checkups are conducted per person, including anthropometric inspection, blood sample analysis, urine sample analysis, physical function tests, genome analysis, microbiome analysis, and lifestyle-related factor surveys. Of these IHPP participants, 270 who provided informed consent were included in the present study.

In recent years, the extraction of influential factors using machine learning has been widely performed. In particular, Bayesian network (BN) analysis has been introduced as a method for analyzing the causal relationships among health check items [25,26]. In a previous study, health-related data were evaluated using machine learning models [27], and disease prediction models were developed using multi-omics data. However, to the best of our knowledge, there are no studies in which factors affecting antibody production and side effects are extracted using machine learning, or where their causal relationships are explored using BN analysis.

This study aimed to explore lifestyle factors related to antibody production and adverse reactions after mRNA vaccination. Specifically, this study employed a machine learning approach using an extraordinarily rich multi-omics dataset obtained from participants in the IHPP health checkups.

The pandemic can only end after achieving full vaccination worldwide, which minimizes hospitalizations due to SARS-CoV-2 infection. If herd immunity is not achieved through “voluntary” vaccination, government-mandated “compulsory” vaccination will undoubtedly be necessary.

Detailed research to deepen understanding of pathophysiological processes, clarify risk factors, and potentially reduce the incidence of these events carries a crucial responsibility: to foster public confidence in vaccination, thereby supporting government-mandated vaccination [28].

## 2. Materials and Methods

### 2.1. Participants

Inclusion and exclusion criteria were defined prior to participant selection to ensure a clearly characterized study population. The study protocol was approved by the Hirosaki University School of Medicine (approval number 2020-046-5 and 2021-017-9). Of the participants in the 2021 IHPP health checkups, 270 who provided informed consent were included in this study. Blood samples were drawn from participants at the designated hospital (Hirosaki Onsen Yojo Clinic, Hirosaki, Japan) after the second and third mRNA vaccinations. Our original health questionnaire regarding vaccination and its side effects was completed upon blood sample collection (Table S1 (Supplementary Material)). Individuals were eligible for inclusion if they participated in the 2021 IHPP health checkups, provided written informed consent, received both the second and third doses of mRNA COVID-19 vaccines, completed the original vaccination questionnaire, and had blood samples collected at the designated hospital.

**Figure 1:**
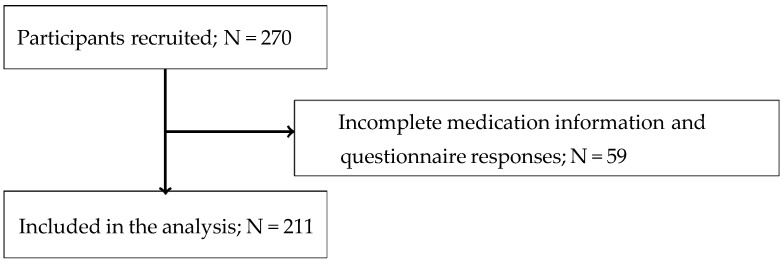
Participant flow.

Participants were excluded from the study if they were taking medications that might affect antibody production (e.g., steroids or immunosuppressants) based on their medical history and medication status in the years before and after the 2021 IHPP health checkups and any condition that could interfere with the interpretation of antibody responses, as judged by the study investigators (e.g., severe chronic illness or immunodeficiency). Fifty-nine participants were excluded due to incomplete medication information or questionnaire responses. The final study population comprised 211 participants (Figure 1).

Age categories were defined as “younger” and “older,” with a threshold of 60 years, which is the typical menopausal age in women. While the average menopausal age for Japanese women is in their 50s, based on the age-related trends of FSH, LH, and free testosterone in both men and women in this cohort, we classified participants as younger or older groups, defining menopause at age 60.

### 2.2. Blood Sample Collection and Questionnaires

**Figure 2:**
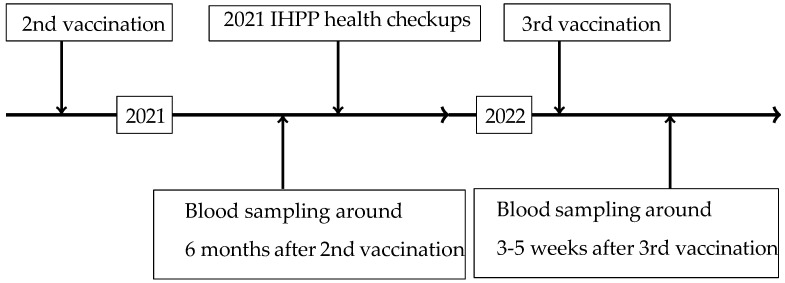
Study protocol and evaluation of SARS-CoV-2 spike protein IgG titers.

In the present study, blood samples were collected six months after the second vaccination and 3–5 weeks after the third vaccination. The time series for the 2021 IHPP health checkups and post-vaccination blood samples is shown in Figure 2.

At the injection sites, the participants were asked to complete a questionnaire regarding the prevalence of exogenous side effects, including pain, redness, swelling, and itching, as well as endogenous side effects, including fatigue, headache, myalgia, arthralgia, chills, nausea, and fever symptoms (“fever” and “feverish”). For fever symptoms, the maximum body temperature (°C) was recorded.

The evaluation of body temperature was classified into two categories, with a threshold of 38.0 °C. Plasma samples were collected using blood collection tubes with a serum separator, which were then centrifuged and analyzed. An ARCHITECT SARS-CoV-2 (Abbott Japan LLC, Tokyo, Japan) was used to detect anti-SARS-CoV-2 spike protein IgG (sIgG) and IgM (sIgM). An ARCTHEC i2000 (Abbott Japan LLC) was used to measure the SARS-CoV-2 nucleocapsid IgG antibody (N-IgG) antibody. SARS-CoV-2 infection status was assessed by measuring N-IgG, and the study proceeded after confirming that all 211 participants were uninfected.

Blood sample analyses were performed by the LSI Medience Corporation (Tokyo, Japan). The actual SARS-CoV-2 sIgG values were used as antibody titers. The quantitative results of the antibody titers are presented as standardized units (AU/mL).

### 2.3. IHPP Health Checkup Data

For the 2021 IHPP health checkups, 3745 items, including health status from the health status questionnaires, dietary habits (especially vitamin and carotenoid intake) from a food frequency questionnaire (brief self-administered diet history questionnaire [BDHQ], DHQ Support Center, Tokyo, Japan), cardio ankle vascular index using a blood pressure pulse wave inspection device (VS-2000, Fukuda Denshi Co., Ltd., Tokyo, Japan), body composition from a body composition analyzer (Inbody710, InBody Co., Ltd., Seoul, Republic of Korea), visceral fat area from a visceral fat meter (EW-FA90, Panasonic Corp., Tokyo, Japan), telomere G-tail length, oral and gut bacterial flora, and other items (Table S2 (Supplementary Material)) were collected. The obtained data were organized as research data by the Hirosaki University Data Management Committee, and this study utilized them for analysis.

### 2.4. Bayesian Network (BN) Analysis

Before the BN analysis, screening was performed to extract factors associated with antibody production and adverse reaction symptoms from the various health checkup items [29,30]. Several machine learning regression models with antibody titers and adverse reactions as objective variables and questionnaire results and multi-omics data as explanatory variables were constructed [31,32]. The machine learning models include ridge regression model, light gradient boosting on elastic net prediction model, Keras slim residual neural network regression model, and elastic-net model. Then, feature impacts, representing the importance of specific health checkup items with high predictive accuracy were obtained [33]. Table S3 shows approximately 100 influencing factors extracted from a total of 3745 items.

The dataset was randomly divided into training (80%) and test (20%) data. A prediction model was built using the training data, and its validity was evaluated using the test data. A series of random divisions of the dataset, model creation including hyperparameter tuning, and model evaluation were repeated ten times to overcome the sampling error. Model evaluation was performed by calculating the root mean squared error and the area under the receiver operating characteristic curve.

BN analysis was performed to evaluate the network of items related to antibody production. The most important feature is determining the interrelationships between variables and representing them as a phylogenetic network diagram [34]. A BN examines the causal relationships among variables and measures the strength of causality through conditional probabilities, which quantify the likelihood of other events occurring when a specific event transpires. The visualization of the network topology in this study adopted a combination of the greedy hill-climbing algorithm and the bootstrap method. Using this algorithm, the local optimal network structure for the target dataset was calculated, and the final network structure was determined by evaluating its stability using 1000 independent bootstrap runs.

After BN analysis, the derived relationships among the variables were determined as a list of edges connecting parents and children. The influence of the interactions between these variables was calculated as a positive or negative relationship. The stability of the results was evaluated using bootstrap probability. Bootstrap probability was defined as the occurrence rate of an edge and was used as an index of strength between two factors, ranging from 0 to 1 [35]. The edge gain value was also calculated as the impact of each edge and was used in the network assessment [36]. In some cases, the edge gains were not calculated correctly as they exhibited large negative values; therefore, these results were excluded. When these outliers were calculated, they were considered unavailable [34]. Thus, edge gain was also used as a criterion to determine the strength of the branch of interest.

### 2.5. Statistical Analysis

Analytically measured values were reported as numerical values, and categorical items such as responses to the questionnaire were reported in Boolean form. In particular, measured values were used for indigenous bacteria in the tongue coating and gut bacterial flora. For special bacteria such as opportunistic bacteria, Boolean data were transformed according to the percentage of bacteria present. For the influencing factor analysis, the antibody titers were converted to robust z-scores, and the side effects from the health questionnaire were converted to a Boolean form.

BN analyses were performed using INGOR which is our originally developed software [34,37] on the SHIROKANE supercomputer system at the Human Genome Center, Institute of Medical Science, University of Tokyo, and the IWAKI supercomputer system at the Graduate School of Medicine, Hirosaki University. The analysis software JMP Pro (Versuib 16. SAS Institute Inc., Tokyo, Japan), IBM SPSS 28.0. 1 Statistics Base (IBM Japan, Ltd., Tokyo, Japan), and R (version 4.3.1) were used for statistical analyses, while Data Robot^®^ (Data Robot, Inc., Boston, MA, USA) was used for the screening. All statistical tests were two-tailed, with a significance level at 0.05.

## 3. Results

### 3.1. Participants and Their Series of Vaccines

Table 1 presents the characteristics of the participants. Notably, participants living in the Iwaki district had a higher visceral fat area than the national average [38]. Residents in Hirosaki City, including the Iwaki district, were recommended to receive the same type of vaccines in the first and second vaccinations, and the third vaccine of either Comirnaty or Spikevax and was of her/his choice. Thus, the combinations of first, second and third vaccine types was any of the following: Comirnaty–Comirnaty–Comirnaty, Comirnaty–Comirnaty–Spikevax, Spikevax–Spikevax–Comirnaty, or Spikevax–Spikevax–Spikevax. The most common combinations were Comirnaty–Comirnaty–Comirnaty and Comirnaty–Comirnaty–Spikevax; therefore, we focused on these two vaccine combinations in subsequent statistical analyses.

### 3.2. sIgG Levels and Side Effects After the Third Vaccination

Table 2 shows an analysis of variance table for sIgG after third vaccination. The following statistical model was used:

log(*sIgG*) = *µ* + *Sex* + *AgeGroup* + *VacType*
+ *Sex* × *AgeGroup* + *Sex* × *VacType* + *AgeGroup* × *VacType* + *ε*, (1)

where *µ* is the overall mean and *ε* is a residual following a normal distribution. *AgeGroup* is a categorical variable of “Younger” and “Older” levels, and *VacType* is a categorical variable of “Comirnaty–Comirnaty–Comirnaty” and “Comirnaty–Comirnaty–Spikevax” levels. The distribution of residuals did not reveal any significant skewness. Regarding multicollinearity, we confirmed that the variance inflation factor was less than 5 for all terms.

The interaction between the vaccine type and age group was significant (*p* = 0.007). These results suggest the necessity for stratification by age group and vaccine type.

Table 3 shows effects of series of vaccine type stratified by age group. The mean antibody titers showed a statistically significant difference among the vaccine combinations in the younger group (*p* = 0.001); however, no statistically significant difference was observed in the older group. Although no statistical difference in side effects was observed between vaccine combinations in the younger and older groups, a statistical difference between “fever and feverish” was observed across vaccine combinations in the younger group (*p* = 0.025), but not in the older group. This suggests that there was no significant difference among the vaccine combinations in fever severity between the younger and older groups.

### 3.3. Relationship Between Side Effects and sIgG

Table 4 shows results of logistic regression analysis for onset of side effects and fever, and severity of fever. The side effects are defined as any subjective symptoms of exogenous side effects including pain, redness, swelling, and itching, and endogenous side effects, including fatigue, headache, myalgia, arthralgia, chills, nausea, and fever and feverish. The severity of fever is an ordered categorical variable of less than 38 °C, equal to 38 to less than 39 °C, and equal to or greater than 39 °C. The explanatory variables were main effects of concentration of sIgG (*sIgG*), sex (*Sex*), age group (*AgeGroup*), vaccine type combination (*VacType*), and interaction of *Sex* and *AgeGroup*, *Sex* and *VacType*, and *VacType* and *AgeGroup*.

The *sIgG* significantly increased the odd ratio of fever and feverish, and severity of fever (*p* = 0.004 and *p* = 0.002, respectively). As for side effects, *AgeGroup* was shown significantly low odds ratio in older group (*p* = 0.004), and *Sex* was shown significantly low odds ratio in male group (*p* < 0.001). As for fever and feverish, *AgeGroup* was shown significantly low odd ratio in older group (*p* = 0.010), and the interaction of *Sex* and *AgeGroup* was shown significantly low odds ratio in male and older group (*p* = 0.027). As for severity of fever, *AgeGroup* was shown significantly low odds ratio in older group (*p* < 0.001). The *VacType* and its interaction with *Sex* nor *AgeGroup* were not shown any significance.

### 3.4. BN Analysis

Factors influencing antibody titers and adverse reaction symptoms calculated from BN analysis are shown in Table 5 (a; Spearman’s rank correlation coefficient, b; Exact Wilcoxon Rank-Sum Test) and Table 6 (presence of adverse reactions and fever symptoms). Table 5 indicates that three factors (sIgM, albumin, drinks (green tea: 2–3 cups daily)) positively correlate with antibody titers (sIgG).

Factors associated with the prevalence of adverse reactions included *Nisseriaceae*, Folate, Metabolic compounds (Sarcosine, 2-Oxoisovaleric acid, and Hypoxabtgube), Grip strength (left side), Lymphocyte subset (CD16(+) × CD56(+)), and Luteinizing hormone. Among these, *Nisseriaceae*, Folate, and Luteinizing hormone showed a positive proportional relationship, while the others showed a negative relationship. Factors associated with the prevalence of fever symptoms included Telomere (post) length, sIgG, and Lymphocyte subset (CD4(+) × CD8(−), all of which showed a positive proportional relationship.

### 3.5. A Relationship Between Green Tea Intake and Antibody Titers

To investigate the relationship between green tea intake (g/day) and antibody titers, participants were divided into tertiles based on antibody titers (Low group, ≤15,100 AU/mL; Medium group, 15,800–29,400 AU/mL; and High group, ≥29,400 AU/mL).

**Figure 3:**
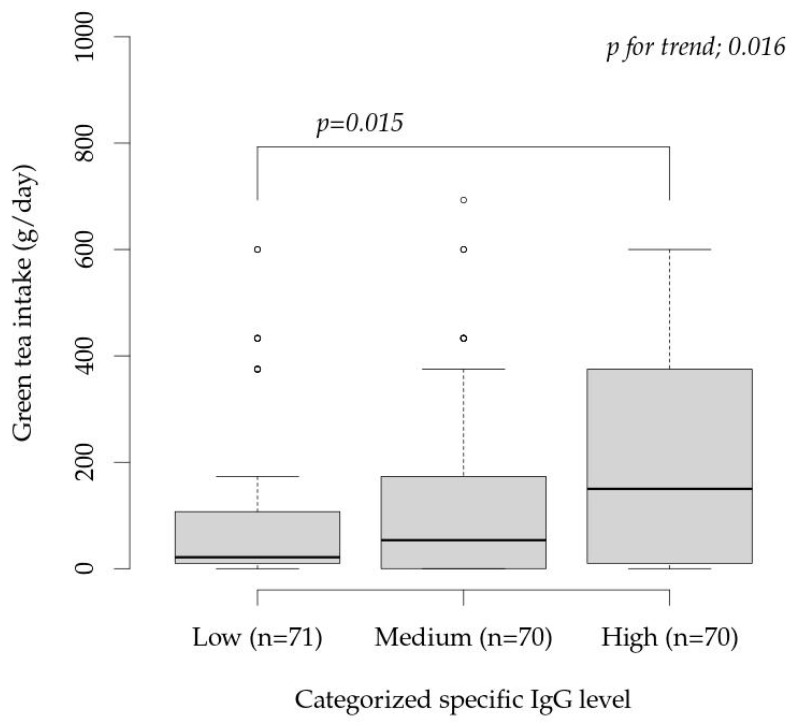
Comparison of green tea intake (g/day) between groups. The subjects were divided into tertiles based on antibody titers. “*p* for trend” is result of Jonckheere–Terpstra test. The comparison between groups was made with Wilcoxon rank-sum test without adjustment for the multiple comparisons.

Figure 3 shows the comparison between groups. The Jonckheere–Terpsta test showed significant increasing trend of green tea intake by sIgG (*p* for trend = 0.016). The Wilcoxon rank-sum test showed a significant difference (*p* = 0.015) between Low group and High group, indicating that green tea intake was significantly higher in the group with a high antibody titer. The mean daily green tea intake (Median (25% tile–75% tile)) (g/day) in the Low, Medium, and High groups were 21.4 (10.0–107.1), 53.6 (0.0–173.2), and 150.0 (10.0–375.0), respectively.

## 4. Discussion

In this study, we examined the impact of vaccination in the two groups with the highest number of participants (Comirnaty–Comirnaty–Comirnaty and Comirnaty–Comirnaty–Spikevax) (Table 1). From the analysis of variance table for the statistical model with the outcome variable being the antibody titer after the third vaccination (Table 2), the interaction between the type of vaccine administered for the third dose and age group showed statistically significant results (*p* = 0.007). Previous studies have reported that when a booster dose is administered, Spikevax is associated with a higher risk of adverse reaction symptoms than Comirnaty [39,40,41]. Because the participants in this study received only the third dose of a different vaccine type, we found that the risk of adverse reaction symptoms varied depending on the age and type of vaccine administered for the third dose.

Regarding antibody production patterns in the younger age group (≤60 years; Table 3), a significant difference was observed based on the type of vaccine used for the third dose (Comirnaty < Spikevax, *p* = 0.001). Analysis of adverse reaction symptoms also confirmed a similar significant difference in the proportion of patients experiencing fever symptoms (*p* = 0.025). However, no significant difference was observed in fever severity (*p* > 0.999). This suggests that the presence or absence of fever symptoms may contribute to the higher antibody titers observed in the group receiving Spikevax as the third dose. In contrast, no statistically significant differences based on the vaccine type were observed in the older group (*p* = 0.931).

Adverse reactions following mRNA vaccination are induced by LNPs, which serve as drug delivery systems for mRNA and their carrier adjuvants [42,43]. Findings regarding the impact of young and elderly participants on antiviral pathways and immune function have demonstrated that empty LNPs (eLNPs), which are components of mRNA-based vaccines, induce maturation and cytokine production in monocyte-derived dendritic cells. However, age-specific differences in the maturation and activation of these cells have also been observed, suggesting its potential role in the overall reduction in antiviral and vaccine responses. The most commonly used COVID-19 adjuvants include β-defensins, aluminum salts, and matrix proteins. These adjuvants activate innate immune signaling, enhance antigen-presenting cell activity, and trigger inflammasome activation.

Table 3 indicates that the risk of fever symptoms varied among younger individuals depending on the type of vaccine administered. In contrast, among older individuals, there were no differences in adverse reactions, fever symptoms, or fever severity based on the vaccine type. One reason considered for this is that, while the risk of adjuvant-related adverse reactions occurs regardless of sex or age, the responsiveness to eLNP treatment is age-dependent, leading to differences in the risk of adverse reaction symptoms accompanied by fever across age groups.

The logistic regression analysis of adverse reactions in Table 4 used the Male Younger Comirnaty–Comirnaty–Comirnaty group as the reference. Variables with statistically significant differences based on the 95% confidence intervals of the odds ratios (ORs) are “Side effects” (OR = 0.14, [95% CI; 0.02, 0.86]) and “Fever and Feverish” (OR = 0.14, [95%CI; 0.02, 0.86]) in the case of the Male Older Comirnaty–Comirnaty–Comirnaty group. Both had ORs < 1, indicating that the group Male Older Comirnaty–Comirnaty–Comirnaty had a statistically significantly lower risk of “Side effects” and “Fever and Feverish” compared to the Male Younger Comirnaty–Comirnaty–Comirnaty group. No other statistically significant differences in the risk of occurrence of adverse reaction symptoms were observed. In other words, when receiving Spikevax, which is associated with a higher incidence of adverse reactions, the risk of adverse reactions was similar regardless of sex or age. However, when receiving Comirnaty, which is associated with milder adverse reaction symptoms, the age category affects the risk of adverse reaction symptoms in males.

Reports indicate that for several vaccines, including SARS-CoV-2 vaccines, females have a higher risk of adverse reactions and higher antibody production than males [44]. Furthermore, free testosterone has been reported to suppress the action of inflammatory cytokines, is strongly associated with disease severity in patients with COVID-19, and exhibits a higher inhibitory effect on the SARS-CoV-2 main protease than progesterone [45,46,47,48]. Additionally, antibody production patterns decline with increasing age and are associated with adverse reactions [49,50]. The observed differences in adverse reaction risk according to sex and age following Comirnaty vaccination suggest that hormones related to inflammatory cytokines may influence these outcomes. Furthermore, since fever symptoms following Spikevax vaccination were associated with a tendency toward increased antibody production (Table 3), it was considered that the higher risk of fever in younger age groups likely impacted antibody production. A comparison of the ORs for sex differences in the younger age group revealed no significant differences. Therefore, the risk of fever symptoms did not show a significant sex difference.

Previous reports have indicated that visceral adipocytes release proinflammatory cytokines that promote the activation and proliferation of immune cells [51,52]. Furthermore, visceral fat area (VFA) is associated with an increased risk of severe disease in COVID-19 patients [53,54] because angiotensin-converting enzyme II (ACE2), the receptor for SARS-CoV-2 [55], is more highly expressed in visceral adipose tissue (VAT) than in subcutaneous fat. In other words, infected patients with greater VAT accumulation and a larger VFA have a higher risk of developing severe disease. While the participants in this study received mRNA vaccines rather than contracting COVID-19, the increased expression of ACE2 originating from visceral fat is expected to be similar due to infection or vaccination.

Among men, both the younger and older age groups had average VFA levels exceeding 100 cm^2^ (107 cm^2^ for younger men and 115 cm^2^ for older men), with obesity levels being slightly higher in the older group. In contrast, the VFA of women was higher in the older group (71 cm^2^) than in the younger group (59 cm^2^) but did not reach the levels seen in men. Calculation of the correlation coefficient between the VFA and antibody titers from the IHPP data revealed statistical significance only in the younger group, with Spearman’s correlation coefficient of 0.226 (*p* = 0.020). Therefore, a large VFA is considered a possible cause of the higher incidence of adverse reactions and fever in younger males and the associated high antibody titers.

Statistical evaluation of the questionnaire results and antibody titers revealed that (1) age and sex at vaccination were the primary factors influencing post-vaccination antibody production patterns; (2) among younger individuals, the group receiving Spikevax had a higher risk of fever accompanied by higher antibody titers than the group receiving Comirnaty as the third dose (which exhibited milder adverse reactions than Spikevax); and (3) elderly males have a significantly lower risk of adverse reactions than younger individuals. Based on these results, it was considered that the immune response to LNP, the foundation of mRNA vaccines, differs by age, and that the risk of adverse reactions varies due to hormonal influences related to sex differences, both of which affect antibody production patterns. Furthermore, in younger individuals with visceral fat obesity, the presence of adverse reactions and antibody production patterns has been suggested to involve ACE2 expressed in the visceral fat.

We then extracted items with high-impact factors and calculated their relationships using BN analysis. We set the antibody titers after vaccination and the presence or absence of adverse reactions or fever symptoms as dependent variables and identified items showing statistically significant differences (Table 5 and Table 6).

sIgM and albumin levels were found to have statistically significant positive correlations with the antibody titers. sIgM, an immunoglobulin produced by B cells, plays a major role in the initial phase of infection, before sIgG antibodies become active. Furthermore, albumin, a plasma protein, is reported to be an effective and efficient predictor of the viral shedding period owing to its strong binding affinity, which allows it to bind to various substances [56]. Therefore, it is understandable that these two factors significantly influence antibody production patterns.

Green tea has been reported to be effective in improving periodontal disease, suppressing influenza onset [57], weakening viral infections [58], and improving nasal symptoms [59], suggesting that it may also influence COVID-19 transmission pathways. While the BDHQ used in this study did not examine the type of green tea consumed or the extraction method, the results suggested that green tea may influence antibody production patterns in COVID-19. The relationship between green tea intake and antibody production patterns shown in Figure 3 clearly distinguishes the green tea intake levels of participants with low antibody production patterns from those with high antibody production patterns, confirming that green tea intake significantly influences antibody production patterns.

Factors associated with the presence or absence of adverse reactions included bacterial flora in the tongue coating (family) *Neisseriaceae*, Folate, sarcosine, 2-oxoisovaleric acid, left handgrip strength, hypoxanthine, CD16(+)CD56(+) cell subsets, and luteinizing hormone (LH). Commensal *Neisseria* species have been suggested to modulate innate immune responses and cytokine production in experimental infection models and may be associated with pathophysiological processes during SARS-CoV-2 infection [60,61]. Folate has been reported to have antiviral effects and it acts on the SARS-CoV-2 nucleocapsid protein [62]. Additionally, while there was no difference in LH levels among women of childbearing age following SARS-CoV-2 vaccination, studies across all sexes and age groups have reported that high serum LH levels may increase the clinical severity of COVID-19 [63].

The bacterial flora of the tongue coating (family) *Neisseriaceae*, Folate, and LH showed statistically significant positive correlations with the presence or absence of adverse reactions. In other words, compared to participants with a low risk of adverse reaction symptoms, those with a high risk tended to have higher levels of these factors. COVID-19 is a systemic disease that significantly affects cellular metabolism. Sarcosine, a plasma metabolome metabolite, significantly increases the number of leukocytes that exhibit IgG memory [64]. Similarly, 2-oxoisovaleric acid levels have been reported to increase in severe COVID-19 cases, indicating its potential as an infection marker [65]. Furthermore, hypoxanthine has been reported to play a role in inflammation and coagulation disorders in COVID-19 [66]. Although the metabolome pathways directly involved differ, these substances exert an influence on participants at a high risk of adverse reactions. The CD16(+)CD56(+) lymphocyte subset, which indicate natural killer (NK) cells, have been reported to correlate with lower spike-specific IgG levels after booster vaccination in groups with higher pre-vaccination NK cell counts [67]. Thus, low NK cell counts can be considered an indicator of participants with lower adverse reaction risk. Grip strength [68], a representative indicator of overall muscle strength, is significantly correlated with the presence or absence of adverse reactions. Because muscle strength involves various factors, muscle strength indicators alone cannot explain the causal relationship with adverse reaction risk. The results calculated in this study showed that participants with high adverse reaction risk had low muscle strength, while those with a low risk had high muscle strength. This suggests that the nervous system and muscle quality, not only muscle mass, are involved in adverse reaction risk.

Factors associated with the presence or absence of fever symptoms were telomere length, antibody titer, and the CD4(+):CD8(−) ratio. Telomeres are genome-protective structures located at the chromosome ends. They shorten after each DNA replication cycle, associating them in aging mechanisms. Previous studies reported shortened telomere lengths in patients with COVID-19 [69]. As this study recreated a simulated infection state using mRNA vaccines, the risk of adverse reaction symptoms after vaccination is also thought to have some relevance. Furthermore, it is known that severe adverse reactions, including fever, can affect antibody production patterns [70]. In addition, the CD4(+) × CD8(−) parameter, a lymphocyte subset test item, indicates CD4-positive T cells. These cells induce the functional expression of other T cells, promote B cell differentiation and maturation, and induce antibody production. The results in Table 3 and Table 4 also indicate that fever symptoms affect antibody production patterns. Because all three calculated parameters were directly proportional to the presence of fever symptoms, longer telomere length, higher antibody production patterns, and greater numbers of CD4-positive T cells are indicators of participants at a higher risk of fever symptoms.

This study has several limitations. First, the data are based on a specific geographic region and racial composition, requiring caution when generalizing to other areas. Second, some variables relied on self-report, potentially introducing memory bias or social desirability bias. Third, while the sample size was adequate for the primary analyses, the study design inherently limits inferences of causality and the complete exclusion of unmeasured confounders. Despite these considerations, this study provides important insights into factors influencing antibody production patterns and adverse reaction symptoms.

## 5. Conclusions

Statistical evaluation revealed that the antibody production pattern following vaccination is influenced by age-related differences in immune responses to LNP, the core component of mRNA vaccines and sex-related hormonal effects that alter the risk of adverse reaction symptoms. Furthermore, there was a significant correlation between the VFA and antibody production patterns in men with visceral fat at obesity levels. These results corroborate existing reports, confirming that the questionnaire data on antibody production patterns and adverse reaction symptoms post-vaccination in this study constituted a dataset that was sufficiently robust for factor analysis using machine learning. Furthermore, our statistical evaluation clearly demonstrated that fever symptoms influenced antibody production patterns, yielding highly interesting results. Using this dataset and the IHPP big data to explore the factors influencing antibody titers and the presence/absence of adverse reaction symptoms yielded the following items:

- Factors influencing antibody titers: sIgM, ALB, beverage (green tea);
- Factors influencing the presence/absence of adverse reaction symptoms: tongue coating bacterial flora (family *Neisseriaceae*), Folic acid, sarcosine, 2-oxoisovaleric acid, left grip strength, hypoxanthine, CD16(+) × CD56(+), LH;
- Factors influencing the presence of fever symptoms: telomere length, antibody titer, CD4(+) × CD8(−).

The machine learning approach was executed without issues, and the computational results showed no significant deviations, indicating the above factors are valid findings. The calculation of sIgM and ALB, which are factors involved in initial infection and viral shedding, as factors influencing antibody titer confirms the accuracy of the computational results. In addition, comparing the low and high antibody titers groups showed a significant difference in green tea consumption per day. The finding that consuming green tea had an impact was a highly intriguing result of this study. Further studies examining intake levels and antibody titer fluctuations are considered necessary to clarify the effects of green tea consumption.

Factors increasing the risk of adverse reactions included the blood NK cell count and muscle quality in the arm receiving the vaccine. LH was also calculated as a directly proportional influencing factor, providing results supporting the involvement of hormonal substances in the risk of adverse reactions. Furthermore, plasma metabolome metabolite concentration, tongue coating bacterial colonization, and folate intake were also found to be factors influencing adverse reaction risk. Additionally, the characteristics of participants at risk for fever symptoms included longer telomere length, higher antibody production patterns, and greater numbers of CD4-positive T cells.

Overall, this study enabled the calculation of factors influencing antibody production and the occurrence of adverse reactions following SARS-CoV-2 vaccination by analyzing influencing factors from big data using a machine learning approach. Further investigation of these calculated influencing factors is expected to clarify evidence for new vaccine development and identify lifestyle and dietary habits that enhance vaccine efficacy.

Vaccination should be recognized as a foundational component of public health. Beyond the critical role of COVID-19 vaccines, immunization has consistently served as one of the most effective strategies for preventing a wide spectrum of infectious diseases. Decades of global evidence demonstrate that vaccines substantially reduce morbidity, mortality, and healthcare burden, while contributing to herd immunity and protecting vulnerable populations. Emphasizing this broader significance of vaccination provides essential context for interpreting the role of COVID-19 vaccines within the continuum of infectious disease prevention.

## Figures and Tables

**Table 1:** Participants’ anthropometric characteristics and their vaccine series combinations.

Characteristic	Younger, N = 106			Older, N = 105		
		Male	Female		Male	Female
	n	n = 30	n = 76	n	n = 46	n = 59
Age (y)	106	47 (9)	46 (10)	105	70.3 (5.6)	67.9 (5.0)
Height (cm)	106	172 (6)	158 (5)	105	167 (6)	154 (5)
Weight (kg)	106	72 (7)	56 (9)	105	66 (9)	54 (7)
Body mass index (kg/m^2^)	106	24.21 (2.85)	22.27 (3.39)	105	23.82 (2.48)	22.71 (3.07)
Visceral fat area (cm^2^)	106	107 (45)	59 (31)	104	115 (42)	71 (27)
Vaccine combination	104			104		
Comirnaty–Comirnaty–Comirnaty		8 (29%)	40 (53%)		21 (46%)	24 (41%)
Comirnaty–Comirnaty–Spikevax		17 (61%)	31 (41%)		25 (54%)	33 (57%)
Spikevax–Spikevax–Comirnaty		1 (3.6%)	0 (0%)		0 (0%)	0 (0%)
Spikevax–Spikevax–Spikevax		2 (7.1%)	5 (6.6%)		0 (0%)	1 (1.7%)

Mean (SD); n (%); younger is defined as ≤60 years old.

**Table 2:** Analysis of variance table for sIgG after the third vaccination.

	Sum of Squares	Degree of Freedom	F-Value	*p*-Value
Sex	0.063	1	0.168	0.682
VacType	1.287	1	3.435	0.065
AgeGroup	1.110	1	2.962	0.087
Sex:VacType	0.171	1	0.455	0.501
VacType:AgeGroup	2.818	1	7.520	0.007
Sex:AgeGroup	1.120	1	2.989	0.085
Residuals	71.945	192		

The concentration of sIgG was logarithmically transformed.

**Table 3:** Effects of series of vaccine type stratified by age group.

	Comirnaty–Comirnaty–Comirnaty	Comirnaty–Comirnaty–Spikevax	*p*-Value ^2^
**Younger**			
sIgG			
Median (Q1, Q3)	15,450 (9200, 23,650)	26,150 (15,350, 33,700)	0.001
Side effects ^1^			>0.999
Negative	7 (15%)	6 (13%)	
Positive	41 (85%)	42 (88%)	
Fever and feverish ^1^			0.025
Negative	21 (51%)	11 (26%)	
Positive	20 (49%)	31 (74%)	
Missing	7	6	
Severity of fever ^1^			0.925
<38	9 (45%)	14 (45%)	
38–39	9 (45%)	12 (39%)	
>39	2 (10%)	5 (16%)	
Missing	28	17	
**Older**			
sIgG			
Median (Q1, Q3)	20,900 (14,600, 33,200)	23,250 (13,100, 34,600)	0.931
Side effects ^1^			0.298
Negative	18 (40%)	17 (29%)	
Positive	27 (60%)	41 (71%)	
Fever and feverish ^1^			>0.999
Negative	14 (52%)	22 (54%)	
Positive	13 (48%)	19 (46%)	
Missing	18	17	
Severity of fever ^1^			0.704
<38	10 (77%)	13 (68%)	
38–39	3 (23%)	6 (32%)	
39<	0 (0%)	0 (0%)	
Missing	32	39	

^1^ n (%). ^2^ Wilcoxon rank-sum test; Fisher’s exact test.

**Table 4:** Summary table for the results of the logistic regression analyses for side effects, fever and feverish, and severity of fever.

Group	Side Effects		Fever and Feverish		Severity of Fever	
	OR	95% CI	OR	95% CI	OR	95% CI
(Female, Younger, Comirnaty–Comirnaty–Comirnaty)	1.50	0.31, 7.18	1.08	0.26, 4.54	1.86	0.23, 15.18
(Male, Older, Comirnaty–Comirnaty–Comirnaty)	0.14	0.03, 0.69	0.14	0.02, 0.86	0.05	0.00, 1.23
(Female, Older, Comirnaty–Comirnaty–Comirnaty)	0.67	0.10, 4.46	1.02	0.19, 5.56	0.17	0.02, 1.85
(Male, Younger, Comirnaty–Comirnaty–Spikevax)	0.75	0.15, 3.86	4.28	0.75, 24.30	1.62	0.17, 15.37
(Female, Younger, Comirnaty–Comirnaty–Spikevax)	2.36	0.28, 19.90	1.87	0.37, 9.35	1.15	0.14, 9.40
(Male, Older, Comirnaty–Comirnaty–Spikevax)	0.18	0.03, 1.07	0.29	0.04, 2.01	0.24	0.01, 5.32
(Female, Older, Comirnaty–Comirnaty–Spikevax)	1.76	0.35, 8.78	0.85	0.19, 3.76	0.33	0.04, 2.95

**Table 5:** The influencing factors identified from BN analysis calculated to have statistically significant differences from the (a) Spearman’s rank correlation coefficient and (b) Exact Wilcoxon rank-sum test.

**(a) Spearman’s Rank Correlation Coefficient**					
**Bayesian Network Analysis**			**Comparison of Perception of Antibody Titer**		
**To**	**From**	**Probability**	**Spearman’s Rank Correlation Coefficient**		
			**Rs**	***p*** **Value**	
sIgG	sIgM (index/mL)	0.88	0.398	<0.001	
	Albumin (g/dL)	0.17	0.165	0.016	
**(b) Exact Wilcoxon rank-sum test**					
**Bayesian network analysis**			**Comparison of perception of antibody titer**		
**To**	**From**	**Probability**	**FALSE**	**TRUE**	**Exact Wilcoxon rank-sum test** ***p*** **value**
			**Median [Q1, Q3]**	**Median [Q1, Q3]**	
sIgG	Drinks (green tea: 2–3 cups daily)	0.580	−0.121 [−0.584, 0.681]	0.558 [−0.235, 1.401]	<0.001

sIgG data were analyzed using normalized values.

**Table 6:** Influencing factors for “side effect” and “fever and feverish” identified via Bayesian network analysis and calculated to have statistically significant differences (Exact Wilcoxon rank-sum test).

Bayesian Network Analysis			Comparison for Perception of Adverse Reactions		
From	To	Probability	FALSE	TRUE	Exact Wilcoxon Rank-Sum Test; *p* Value
			Median [Q1, Q3]	Median [Q1, Q3]	
Side effect	*Neisseriaceae*	0.069	719.21 [336.06, 1554.6]	1066.9 [426.02, 1966.0]	0.053
	Folate (μg/day)	0.216	1360.0 [1337.5, 1391.3]	1390.0 [1364.4, 1400.0]	<0.001
	Plasma sarcosine for metabolic compounds (Quantitative values)	0.104	3.10 [2.47, 3.83]	2.56 [2.01, 3.16]	0.001
	Plasma 2-oxoisovaleric acid for metabolic compounds (Quantitative values)	0.179	14.99 [12.84, 16.91]	13.52 [11.82, 15.45]	0.005
	Grip strength left (kgf)	0.421	32.9 [25.4, 40.0]	24.7 [21.6, 31.0]	<0.001
	Plasma hypoxanthine for metabolic compounds (Quantitative values)	0.339	0.89 [0.69, 1.35]	0.77 [0.61, 0.93]	0.005
	Lymphocyte subset: CD16(+) × CD56(+) (%)	0.638	17.0 [11.5, 24.5]	11.5 [8.0, 16.0]	<0.001
	Luteinizing hormone (mIU/mL)	0.335	0.1 [0.1, 4.3]	1.9 [0.1, 17.2]	0.031
Fever and feverish	Telomere post	0.164	180,524.3 [166,180.7, 198,343.3]	187,033.0 [171,527.3, 202,605.7]	0.045
	sIgG (AU/mL)	0.159	−0.18 [−0.57, 0.56]	0.43 [−0.45, 1.15]	0.002
	Lymphocyte subset: CD4(+) × CD8(−) (/μL)	0.144	598.0 [476.0, 754.0]	722.5 [531.5, 903.8]	0.004

sIgG data were analyzed using normalized values.

## Data Availability

Data cannot be shared publicly due to ethical concerns. Data are available from the Hirosaki University COI Institutional Data Access/Ethics Committee to researchers meeting the criteria for data access (contact via e-mail: coi@hirosaki-u.ac.jp). Researchers need to be approved by the research ethics review board of the organization of their affiliations.

## Supplementary Materials

The following supporting information can be downloaded at https://www.mdpi.com/article/10.3390/vaccines14020115/s1. Table S1: ‘Health Questionnaire’ form used in this study. Table S2: Items measured in the Iwaki Health Promotion Project in 2021. Table S3: The feature imapacts used for BN analysis are listed (a: antibody titer, b: presence of adverse reactions, and c: presence of fever symptoms).

## Abbreviations

The following abbreviations are used in this manuscript:

BDHQ	Brief self-administered diet history questionnaire
BN	Bayesian network
IHPP	Iwaki Health Promotion Project
LH	Luteinizing hormone
NK	Natural killer
CI	Confidence interval
OR	Odds ratios
VAT	Visceral adipose tissue
VFA	Visceral fat area
